# Reaching people soon after a traumatic event: an exploratory observational feasibility study of recruitment in the emergency department to deliver a brief behavioral intervention via smartphone to prevent intrusive memories of trauma

**DOI:** 10.1186/s40814-021-00916-x

**Published:** 2021-10-07

**Authors:** Marie Kanstrup, Ann Rudman, Katarina Göransson, Emil Andersson, Klara Olofsdotter Lauri, Emma Rapoport, Linda Sunnergård, Maria Bragesjö, Erik Andersson, Lalitha Iyadurai, Emily A. Holmes

**Affiliations:** 1 Department of Clinical Neuroscience (CNS), K8, Psychology, Karolinska Institutet, 171 77 Stockholm, Sweden; 2 Medical Unit for Medical Psychology, Karolinska University Hospital, Stockholm, Sweden; 3 School of Health and Welfare, Dalarna University, Falun, Sweden; 4 Department of Medicine Solna, Karolinska Institutet, Stockholm, Sweden; 5 Functional Area of Emergency Medicine Huddinge, Karolinska University Hospital, Stockholm, Sweden; 6 Department of Psychiatry, University of Oxford, Oxford, UK; 7 Department of Psychology, Uppsala University, Uppsala, Sweden

**Keywords:** Behavior therapy, Emergency service, Hospital, Feasibility studies, Memory, Patient selection, Psychological trauma, Smartphone, Telemedicine, Mobile applications

## Abstract

**Background:**

The current study explored how to recruit patients soon after a traumatic event, to deliver a novel intervention in a new emergency department in Sweden. This brief behavioral intervention aims to prevent intrusive memories and is delivered soon after trauma in the emergency department. In the UK, it has shown promising results. Traumatic events resulting in admission to the emergency department (e.g., road traffic accidents) may result in subsequent mental health problems such as post-traumatic stress disorder, where intrusive memories of the trauma constitute a core clinical feature. Early interventions that prevent intrusive memories after psychological trauma are lacking.

Specific aims were to explore identification of eligible patients (aim 1), fitting in with emergency department staff routines to deliver the study protocol (aim 2), and using the patients’ own smartphones to deliver intervention/control task (aim 3). Two changes to the previous study were (i) extending the trauma types included (ii) a new control condition, also by smartphone.

**Methods:**

This is an explorative observational study. Data was both analyzed descriptively and using the Framework method.

**Results:**

We identified several possible ways to recruit patients, and establish a sense of embeddedness in the Swedish emergency department context and a positive appreciation from staff. The study protocol was tested with 8 participants. Tasks both in the intervention and control condition were readily delivered via patients’ own smartphones.

**Conclusion:**

Recruitment of patients and smartphone delivery of the intervention indicates initial feasibility. Researcher presence and administration of study procedures was successfully fitted to emergency department routines and well received by staff. Further pilot work is warranted, underscoring the importance of our collaboration between nursing and psychology.

**Supplementary Information:**

The online version contains supplementary material available at 10.1186/s40814-021-00916-x.

## Key messages regarding feasibility

- Previous research has shown that our novel intervention to prevent intrusive memories of trauma was successfully delivered to patients in an emergency department (ED) in the UK after road traffic accidents, and further research is warranted. However, uncertainties existed regarding how to recruit and deliver the intervention in a new ED setting (Sweden) and with a more diverse trauma sample. Thus, we needed to explore feasibility of recruitment and intervention delivery in this new context, including access to participants and fitting in with ED staff routines.
- Key feasibility findings include that recruitment of patients and delivery of study procedures worked well in this new setting. Researcher presence and administration of study procedures could be fitted into ED routines, and ED staff were positive.
- Implications for subsequent pilot work include support for the use of patients’ own smartphones for delivery of intervention and control tasks, the choice of podcast as control task, and the extension of different trauma types eligible for inclusion.

## Introduction

Psychological trauma involves “actual or threatened death, serious injury, or sexual violence” [1] (p. 271), whereas trauma in the medical sense is widely recognized as “an injury caused by extrinsic factor causing threat to life or limb.” Most people (c. 70%) will experience a psychologically traumatic event in their life [2], many of which require medical attention. The emergency department (ED) could be a possible setting for delivery of early interventions that aim to prevent mental health problems after psychological trauma.

After a psychologically traumatic event, patients may suffer mental health difficulties including intrusive memories of the trauma, sleep problems, and eventually post-traumatic stress disorder (PTSD) [3, 4]. Intrusive memories, our current focus, are recurring and involuntary sensory (often visual) memories of the traumatic event. They comprise a core clinical feature in PTSD [1], and are thought central to its development. Intrusions can be associated with strong emotions and functional impairment [1, 5–8]. Due to their central association with other symptoms in the acute phase, they have been suggested as a potential candidate for early interventions [9]. They can be distressing in their own right [10].

A novel intervention approach based on cognitive science [11–13] to reduce the number of intrusive memories has recently been tested in hospital following real-world trauma, after road traffic accidents and after traumatic childbirth (emergency cesarean section, ECS) [14, 15]. It aims to target memory consolidation of traumatic memories using a behavioral intervention including computer game play. The intervention includes a brief trauma reminder and then “Tetris” computer gameplay using mental rotation instructions. The reminder briefly orients the participant to the trauma memory, so that it is in working memory. The gameplay, which has high visuospatial demands, is thought to compete with working memory resources involved in the visual mental image of the trauma [10]. When delivered in the ED in the UK, the intervention was found to reduce the number of intrusive memories in the week after a road traffic accident compared to an attention-placebo control [14]. Another study with women in the maternity ward in Switzerland who had undergone traumatic childbirth also showed reduced number of intrusive memories in the intervention group compared to usual care [15]. In both studies, the gameplay intervention appeared to be acceptable to participants. Importantly, consent rates for taking part were high, as 48% of those approached in the ED and 69% of mothers approached after ECS agreed to participate. This can be compared with 10% in another trial in ED using a more extensive psychological treatment [16]. The gameplay intervention was described as feasible, as it could be delivered in a flexible way, fitted around standard care in the ED, did not require trained mental health professionals for delivery (e.g., potential for future nurse or midwife led administration), was low cost, had no known adverse events, did not require talking in detail about the trauma (which is potentially distressing for some patients), and was low in stigma (a gameplay-based approach may be more appealing than seeing a mental health professional) [14]. The ED staff were positive, indicating that confidence in the intervention was established [14].

Given these promising results from the ED in the UK [14], feasibility work to allow a test of replication and extension is warranted. In prior clinical studies, the cognitive task in the intervention was delivered on game consoles (Nintendo DS), and a written activity log was used as the control condition. In Iyadurai et al. (2018) [14], it was suggested that the device for gameplay could be patients’ own smartphones to reduce the physical devices needed in the hospital and therefore enhance future implementation. The attention placebo used in the prior study controlled for attention and time spent with researcher but did not use the same device as the intervention condition. Exploring a control condition that can be delivered by the same device, i.e., smartphone (“specific factors component control”) [17], is an important next step and would comprise an enhanced placebo control compared to the previous study.

To be able to carry out an RCT in a new ED and country more knowledge about the context is needed. “Key issues of uncertainty” include if it is possible to recruit and deliver the intervention in the planned ED setting (in this case, in Sweden), for example, access to participants, establish a sense of embeddedness of researchers in the ED, understanding local ED routines to fit recruitment and delivery into wait-times and not disrupt, and how to collaborate with ED management and staff. Further, the potential for the hospital to adopt this novel intervention is an important aspect (e.g., flexibility and compatibility with current practice, staff values, and attitudes) [18].

The objective of the present observational study was to explore how to deliver the behavioral intervention [14] in a Swedish ED environment. Specific aims were to explore identification of eligible patients (aim 1), fitting in with ED staff routines to deliver the study protocol (aim 2), and using the patients’ own smartphones to deliver intervention/control task (aim 3). Two changes to the previous study were (i) extending the trauma type inclusion criteria (including all trauma types in ED rather than just motor vehicle accidents) and (ii) use of a new enhanced placebo control condition, also delivered on the patient’s own smartphone.

## Methods

### Study design, setting, and sample

This is an exploratory, observational study, guided by identifying a number of key issues of uncertainty of setting up and delivering the behavioral intervention [14, 19]. Qualitative methods were applied before, during, and after testing the study protocol with eight participants in a university hospital ED in Sweden. We observed routines in the ED, the hospital facilities, and interactions with hospital managers and clinical staff. Inclusion criteria for patients were aged 18 or above; experienced or witnessed a traumatic event resulting in admission to the ED; meeting DSM-5 criterion A for PTSD (“Exposed to actual or threatened death, serious injury, or sexual violence”); can be seen in the ED within 6 h after the traumatic event; report memory of the accident; fluent in Swedish; alert and orientated; and have sufficient physical mobility to use their smartphone at the point of taking informed consent. Between February and April 2018, the study procedure was explored with eight participants. All participants provided their written and informed consent prior to participation. The study was approved by the Research Ethics Review Board in Stockholm (dnr 2017/2215-31 and amendment 2018/416-32).

### Study protocol

The study protocol included one face-to-face meeting with patients in the ED on day 1 and remote follow-up assessments (1 week and 1 month). Participants were assigned to either the behavioral intervention (memory cue + cognitive task “Tetris” game play for a minimum of 15 min) or an enhanced attention placebo control task (listening to a Swedish radio podcast or reading a Wikipedia text for the same amount of time as the intervention task, both common smartphone activities). Tasks in both conditions were delivered via patients’ own smartphones. Participants downloaded the appropriate app (iOS or Android) in their own smartphone (intervention group downloaded an official Tetris game, Tetris from Electronic Arts [20]; control group downloaded either Wikipedia Mobile from Wikimedia Foundation or Sveriges Radio Play from Sveriges Radio). All apps were freely available at the time of the study. Further detailed instructions for study procedures were given by the researcher, following a written study protocol. For specific details on intervention delivery, see [21].

The primary outcome was number of intrusive memories (number of times participants experience an involuntary sensory memory from the trauma, e.g., “seeing the car coming” or “the blood”), assessed via self-report in a daily diary during weeks 1 and 5 after the trauma [14].

### Procedure

The preparation phase included discussions with ED management and co-examining ED statistics (trauma types, time of day, trauma admissions each month, etc.). We conducted observations and consulted experienced staff to understand the patient flow and journey (front desk, triage, sequence of procedures, etc.). Adaptations to fit the local ED context and potential differences in training between, e.g., nurses and mental health researchers (e.g., hygiene and safety) that might impact study delivery were explored. We anchored the project with staff by presenting at a staff meeting, including a Q&A session regarding the intervention and potential concerns from staff regarding researcher presence on-site.

We created a training procedure for delivering the intervention and tested this with MSc students in psychology. The training took 1 day and included role play and observation by experienced trauma psychologists. It focused on working with newly traumatized and vulnerable participants, delivery of the intervention protocol and outcome assessments, and how to fit into patient waiting times and not disrupt existing ED routines.

We explored the feasibility of recruitment, delivery, and outcome assessment procedures with eight participants (four control, four intervention, using pre-prepared randomization envelopes).

### Data collection

Researchers collected observational data by writing notes during ED visits and parallel to the recruitment process, regarding how eligible patients were identified, current hospital routines (e.g., organizational procedures, safety routines, staff working routines), and staff attitudes (see Supplementary material, Procedure for collecting observational notes). To ensure high quality in data collection, the researchers gained familiarity with the culture and social setting by spending adequate time to become oriented to the situation, i.e., observing the setting, speaking with and getting to know personnel (prolonged engagement allowing the researcher to obtain more reliable observations). In order to address researcher bias, we also minimized the inclination for the researcher to see what is expected by always having at least 2 researchers/RAs on site who took independent notes with the possibility of comparing observations and having ongoing consensus discussions. Notes were transcribed into Microsoft Word and uploaded to NVivo (version 11 Pro) [22]. The notes amounted to 17 documents, where 127 observations were coded regarding identification of eligible patients and 87 observations were coded regarding staff attitudes. We also collected qualitative feedback from participants in the study via open-ended questions, e.g., “what was it like to play Tetris/read on Wikipedia/listen to a podcast?” and prompts if necessary, e.g., “did it help you to think about something else than the event?”

### Qualitative analyses

We chose the Framework method, used within thematic analyses to manage and analyze qualitative data. It involves a sequence of seven interconnected steps to assist the researcher in structuring the data by moving back and forth across the data until a comprehensible interpretation develops [23]. It is often used in feasibility studies because it is appropriate when the purpose of the analysis is highly focused, and when researchers work with structured questions and aspire to undertake analysis systematically [23, 24]. To minimize bias in the coding, multiple people coded the observational data together, having continuous discussions about data and alternative interpretations (AR, ER, LS). The final coding was also discussed in the full research team, which included ED staff (EmA, KG). See Supplementary material, Qualitative analyses—Framework method for details on analysis and coding.

## Results

### Identification of eligible patients (aim 1)

Researchers spent in total 22 days (on average 6.7 h/day) in the ED during the recruitment phase. A total of 74 patients were screened by the research team, and 12 patients were further assessed for eligibility, from which eight patients were eligible and willing to participate (three females, age *M*_age_ = 51.4, range 31-83 years, age missing for *n* = 1). A majority (75 %) were full-time employed. High school education was completed by *n* = 5 and university education by *n* = 2. Furthermore, six participants were married/co-habitant. Half of the participants (*n* = 4) had parents born in Sweden, *n* = 3 had parents born in Europe, and *n* = 1 had parents born outside of Europe.

Participants had experienced the following: Road traffic accident as car driver, *n* = 2; road traffic accident on bicycle, *n* = 1; road traffic accident, other, *n* = 1; fall from height, *n* = 1; crush injury, *n* = 2; hit by moving object, *n* = 1.

**Fig. 1:**
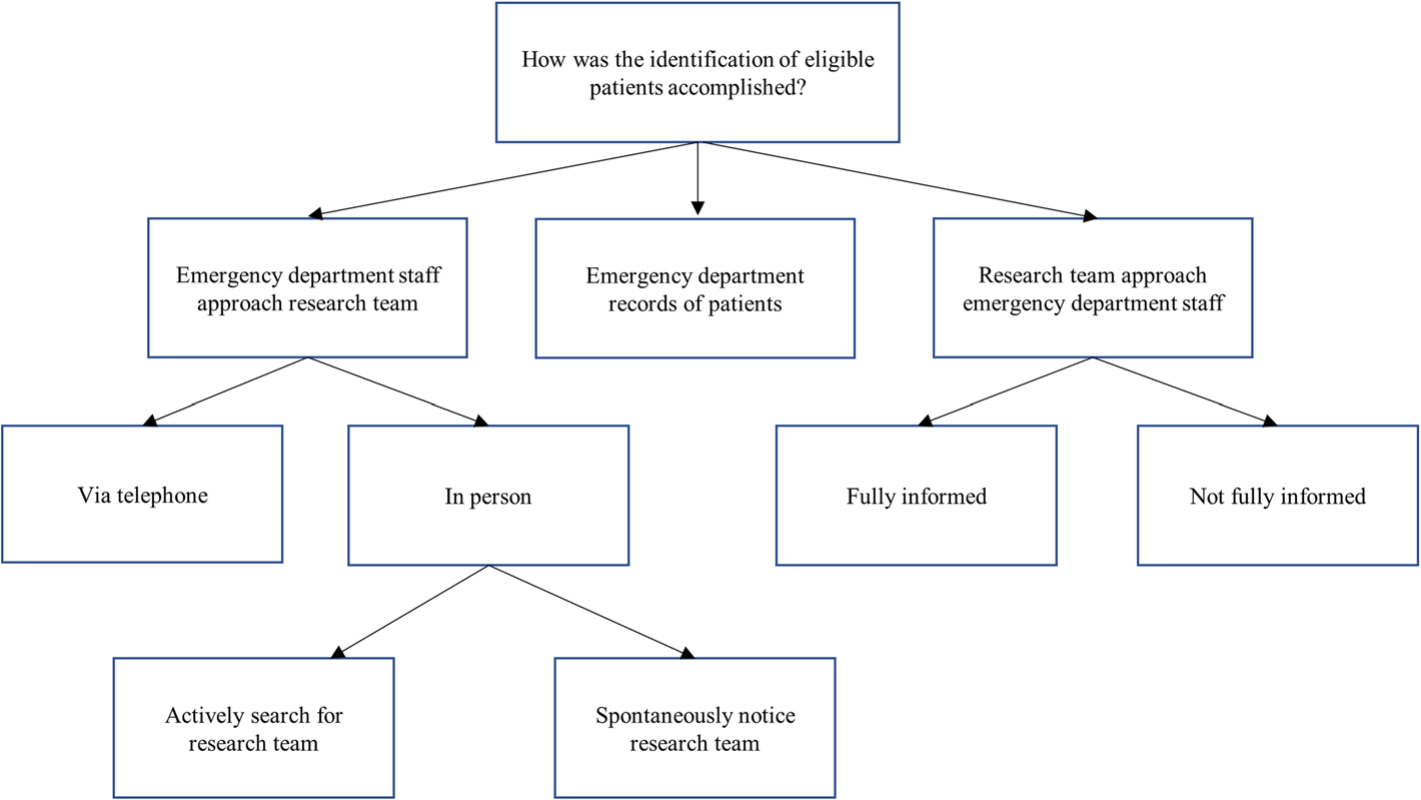
The analytical framework developed regarding the identification of eligible patients (aim 1)

Three possible methods for identification of eligible patients were developed (Fig. 1). The first method was *ED staff approaching the research team* on their own initiative, *via telephone or in person*. To facilitate communication, posters with study telephone numbers and information were placed around the ED, opening up possibilities for staff to detect eligible patients on their own. The presence of the research team in the ED lead to three positive outcomes: Inspiring questions about eligibility criteria from staff, increasing the chance of them identifying eligible patients; facilitating information regarding the study spreading among staff, increasing their involvement in the study and possibly promoting positive attitudes; promoting staff to spontaneously approach the research team about eligible patients. Thus, the research team being on site enhanced the professional relationship and dialog with the ED staff, which turned out to be valuable in the process of identifying eligible patients.

The second method was via *ED electronic medical records* with regards to chief complaints, which turned out to be a time efficient way of identifying patients. It also meant the smallest interruption for ED staff.

The third, and most utilized method for identification of eligible patients was when the *research team approached the ED staff*, and more specifically, the nurse in charge of monitoring the patient flow, with two important factors: First, the relationship with the ED staff, and more specifically if the approached staff member was *fully informed* about the study or not. If the staff member was fully informed, the team only asked a few specific questions, focused on finding eligible patients. If the staff member was *not fully informed*, researchers had to both introduce themselves and inform about the study, meaning that the interaction took more time before eligible patients could be screened. Interactions between staff and researchers were affected by *staff attitudes and commitment, and timing.* A positive *attitude and commitment* from staff regarding the study meant that the team could give more detailed information about inclusion and exclusion criteria. Consequently, screening for eligible participants with the nurse in charge of monitoring the patient flow was more accurate. This implies the importance of both forming a good professional relationship with the staff and promoting positive attitudes regarding the study. *Regarding timing*, it was important not to approach staff members when patient flow was intense or when staff members reported to one another.

### Fitting in with ED staff routines to deliver the study protocol (aim 2)

To understand adaptations needed for delivery in a new ED setting, and establish a sense of embeddedness of the research team in the ED, we discussed with ED management, attended staff meetings, observed staff and patient flow through the ED, and discussed with researchers of previous studies in the ED (L. Iyadurai, K. Porcheret) [4, 14]. It was critical to *fit recruitment and delivery of the intervention into wait-times and not disrupt existing routines in the ED*. This also includes that the *researchers follow patients as needed*, e.g., when transported for X-ray, that researchers are sensitive to staff cues, and flexible in their approach to participants and staff.

An important part of a seamless transition into the local ED was that the *researchers could recruit and move around autonomously*, without the need to disturb staff. This was facilitated by researcher permission to carry swipe-cards for door passage.

The research team needed to *adhere to ED safety and hygiene routines*, e.g., use of hospital clothing, name tags with name and role (researcher), appropriate hand cleaning, in consideration with any patient contact, and access to vaccinations offered to medical staff. This is important as mental health researchers may have less experience following good practice guidelines than hospital staff.

*Facilitating clear communication channels* between ED staff and researchers included study telephones and posters, informing individuals in ED staff about the study repeatedly (given the large number of staff and high staff turnover), weekly email updates from the study team to ED management, and researchers attending daily staff shift change meetings to remind staff about the study.

The ED management and staff expressed that the cooperation with the researchers worked well. The researchers adjusted the study procedure to fit the staffs’ own routines and the patients’ usual care. One reoccurring opinion from staff was that it did not bother them to be approached, since assessing the information the researchers asked for (e.g., whether a potential participant was alert and oriented) was already part of regular work routines. The collective opinion of ED staff was that the research team did not provide any extra burden. They appreciated the team’s effort to help this patient group. Management explicitly expressed that the research teams’ interest in the clinical context was perceived as unique compared to research generally conducted at the clinic.

In the multi-professional team at the ED, several members of the staff were curious and interested in the study, asking detailed questions, e.g., about how the intervention was supposed to work and about the inclusion criteria. Some understood rather quickly what a psychologically traumatic event could be and how it could affect patients. They thought that the intervention approach was promising and asked questions about the recruitment rate, implying that they were interested and wanted to be involved. No negative attitudes toward this specific study or intervention were expressed. However, one of the staff members expressed some negative attitudes toward studies in general done at the ED, implying that on-going research project in the ED was something which the staff simply had to tolerate since it was a university hospital, and which sometimes added to their workload or changed their routines.

Based on observations and discussions with the staff, it was apparent that the patients while waiting often sought out members of the staff, which staff occasionally found time consuming and bothersome. The staff therefore expressed positive attitudes concerning that patients would have something to do while they waited if they participated in the study.

Management expressed their appreciation regarding that regular ED staff did not have to take responsibility themselves for the recruitment of patients, and that the attempt to establish a sense of embeddedness in the ED, and that adapting the study so as not to interrupting staff or adding to their workload, was exemplary. Management, similar to staff, also raised the fact that many different types of research studies collect data via the ED; however, they frequently take for granted that the ED staff will conduct the study procedures (e.g., recruit patients and take samples) thus ignoring the time it takes to conduct studies.

Finally, the intervention rendered interest and optimism. Staff perceived the interdisciplinary aspects of the project (e.g., psychological science) as very positive and rewarding, mentioning that learning about the project and interacting with researchers gave them new insights into the situation of their patients.

### Using the patients’ own smartphones to deliver intervention/control task (aim 3)

We sought to test the procedures for both the intervention and the control task. Of the eight participants, four were given the intervention, and of these, three participants completed the intervention (one was discharged from the ED before starting the intervention), and four were given the control task (two received the podcast option; two received the Wikipedia option). All participants who tested the study procedures were positive toward a smartphone-based intervention delivered within 6 h after a trauma in the ED, and all participants who were offered to participate agreed. All participants in the control group and all but one participant in the intervention group completed the study procedure in the ED. All participants had brought their smartphone with internet access to the ED. Instructions for downloading and using the apps did not pose any difficulties for participants. Smartphones provided a flexible tool for delivery—their light weight and the fact that participants were familiar with their own devices made it possible to deliver the cognitive task component of the intervention even to participants strapped to a gurney (i.e., with restricted freedom of movement).

For the primary outcome at 1 week, all but one participant returned the intrusive memories diary although some prompting was required; six participants returned the pen-and-paper diary by post, while one participant responded to a phone call in which the researcher retrospectively noted the data. There was missing data for one participant. For the same outcome measure at the 5-week follow-up, six of eight participants completed the intrusive memory diary (three via phone).

## Discussion

We explored how to reach patients in a Swedish ED context soon after a psychologically traumatic event, to deliver a brief behavioral intervention aimed at preventing intrusive memories of trauma. We identified eligible patients (aim 1) in three ways: by ED staff initiative, by patient records, and by researchers prompting staff. Fitting in with ED staff routines (aim 2) included assimilating study procedures to fit into wait-times and on-going care, arranging for researcher autonomy within the ED, observing safety and hygiene routines, and establishing clear communication channels with ED staff. Staff and management attitudes to the study and the research team were positive. As for aim 3, the gameplay component of the behavioral intervention was readily delivered via the patients’ own smartphone (rather than a study-owned device) as were the tasks in the enhanced attention placebo control condition (reading a Wikipedia text/listening to a podcast). These findings provide the groundwork for a future pilot trial.

Changes to the original study [14] included (i) extending the range of trauma types included and (ii) use of a new control condition, also delivered on smartphone, which both worked well. Four of the eight participants included here had experienced other trauma types than road traffic accidents, which illustrates the diversity of trauma types in the ED. Older age (the oldest participant included was 83 years) did not seem to constitute a barrier to participation.

To fit into ED staff routines to deliver the study protocol (aim 2), researchers established a sense of embeddedness in the ED. We observed ED staff in their interaction with patients in the ED, who can be experiencing shock, intense fear, panic, pain, and lack of control, and also positive aspects, e.g., ED staff support and communication [25]. Further, we observed ED routine procedures to adjust the study protocol flexibly around medical care. By getting to know the staff, it was possible to ensure that procedures matched staff values and did not interfere with their work. Staff that had been in contact with the team clearly kept the study in “mind” and reported back to the team when they had information about possible participants, and assisted and helped to determine the right time to approach the patient. Despite inevitable pressure in the ED, and the vast number of employees across shifts, it is notable that researchers did not experience any clashes with staff culture or interference with staff preferences.

Research with ED staff has shown the importance of limiting interruptions in ED that are not related to participant safety, and ensuring that communication with staff is done with consideration to the context, in a way that is not disturbing [26, 27]. As the ED context is a complex system with a high workload, strategies to maintain patient safety are very important [28]. We observed that patients waiting in ED can seek and disturb staff when staff are busy doing other tasks. Engaging patients in a self-administered intervention (such as our intervention protocol) while waiting for usual care in the ED may thus also be helpful for staff. By utilizing patient’s waiting time for medical interventions in ED (e.g., for physician examination/diagnostic testing) to complete a behavioral intervention around psychological trauma related to the patient’s reason for visiting the ED, has the potential to change the perspective of ED waiting time from a passive phase of health care (waiting) to an active phase (undergoing intervention).

The study protocol implementation process was experienced by staff as very new and positive, bringing valuable “new eyes on context.” This is in line with suggestions for good clinical research in emergency medicine [29], such as the usefulness of all staff having full knowledge about a planned study, and the importance of feeding back results and potential benefits.

Using patients’ own smartphones to deliver intervention/control task (aim 3) was new to this study. Though a potential barrier to participation would be lack of smartphone with internet access, no eligible participants were excluded due to this, in line with statistics for Sweden showing that 90% of the population have a smartphone [30]. Participants were positive toward smartphone use and no practical difficulties arose.

Smartphone use allowed us to derive a more closely matched attention placebo control condition than the earlier study [14]. That is, the task in both conditions could be displayed on the same device, both be downloaded as apps, with similar time with researcher [31] and we hypothesized maximizing treatment credibility of the placebo.

We tested two app activities, listening to a Swedish radio podcast and reading Wikipedia texts. Both activities were well received and liked by participants. We noticed participants scrolling and examining photographs in the Wikipedia app rendered this activity more visual than expected. We believe that the visual nature of the intervention (i.e., a visuospatial interference task hypothesized to interfere with images of the trauma by competing for working memory recourses [32, 33]) is a critical ingredient to enhancing its efficacy. That is, the ideal control task would match the intervention on expectation effects, duration, device used, time spent with researcher, and engaging in a cognitive task (but ideally not another visual one). Listening to a podcast is a more common activity to do on the phone (compared to using Wikipedia) and therefore more comparable as a popular activity to a game. Thus, we hypothesized that the podcast would be a more useful alternative in future studies as a control task to the gameplay-based intervention, and chose this option for our subsequent exploratory pilot randomized controlled trial [21] and RCT [34].

We took steps to formalize training procedures for delivering the study protocol. This identified the importance of enhanced focus on assessment of the primary outcome (intrusive memories), explaining terms, and how to return the diary. In addition, we noted that such training should include initial close observation of researchers and continued monitoring throughout the study regarding (1) general study procedures (e.g., informed consent), (2) critical steps in the intervention (e.g., memory reminder cue, Tetris task with mental rotation instructions), and (3) inspection of the primary outcome data upon return. Such formalized training procedures would potentially allow future nurse or midwife led delivery, facilitating data collection for a clinical trial and subsequent implementation in a range of hospital settings.

Limitations of this study include the lack of illustrative verbatim quotes. A larger sample of patients would have allowed more firm conclusions on patient preferences regarding study procedures. Furthermore, our analytical framework was developed after the data collection, and we were unable to report the exact number of patients identified for each of the various methods of identifying patients that emerged in this framework.

Though much of the work reported here is in line with what one would expect as part of any study setup, and part of good practice in doing any patient research across different aspects of health care, we have chosen to report our results from this initial phase separately for three reasons, First, hitherto, to our knowledge, few psychological studies have been conducted in the ED with the aim to prevent mental health problems after trauma—and any adaptations of study procedures to fit in a busy ED are not reported in detail in these papers. Second, our previous study in an ED in UK using this intervention showed promising inclusion rates, with only 67 of 140 approached patients declining to take part [14]. Other studies conducted in the ED with the aim to prevent mental health problems after trauma show a higher proportion of eligible patients declining study participation (e.g., 88% in a psychotherapy trial [16], 58% in a pharmacological trial [35]). Recently, a trial evaluating brief prolonged exposure in the ED soon after trauma was terminated prematurely on basis of major recruitment difficulties including hospital reorganization and difficulties reaching eligible participants soon after trauma [36]. Furthermore, recruitment difficulties were also encountered in a study on brief prolonged exposure soon after rape, here, specifically due to the time criterion [37]. Taken together, this prompted us to carefully explore how to recruit soon after trauma, prior to starting the pilot study in this new ED setting in Sweden [21]. Indeed this exploratory work co-creating ways of recruiting with ED staff is a factor that may have helped the next studies we did in finding a recruitment rate of 88% willing to complete study procedures (that is of those approached with a possible traumatic event, only 14/115 were unwilling to complete study procedures, see Fig. 1 Participant flow, in [21]). Third, with a long-term goal of implementation of our novel intervention at scale, e.g., via nurse-led delivery in the ED, we were particularly interested in understanding procedures for identifying patients in the ED who might benefit from the intervention, and how the procedures were perceived by staff, and to examine the recruitment processes directly.

Following this work, a pilot study is needed to generate sufficient information for estimations of sample size for a subsequent clinical trial [38]. Such a stepwise process is critical, ensuring procedures are “optimised enough” [39].

## Conclusion

ED recruitment of patients and delivery of a brief behavioral intervention via smartphone to prevent intrusive memories after psychological trauma indicates initial feasibility. Study procedures were successfully embedded into waiting times and routines in the ED, and further pilot work is warranted.

## Supplementary Information

**Additional file 1.** Procedure for collecting observational notes. Qualitative analyses - Framework method.

## Data Availability

The datasets during and/or analyzed during the current study are available from the corresponding author on reasonable request.

## References

[1] American Psychiatric Association (2013). Diagnostic and statistical manual of mental disorders: DSM-5. Fifth edition.

[2] Benjet C, Bromet E, Karam EG, Kessler RC, McLaughlin KA, Ruscio AM (2016). The epidemiology of traumatic event exposure worldwide: results from the World Mental Health Survey Consortium. Psychol Med.

[3] Ahl R, Lindgren R, Cao Y, Riddez L, Mohseni S (2017). Risk factors for depression following traumatic injury: an epidemiological study from a Scandinavian trauma center. Injury..

[4] Luik AI, Iyadurai L, Gebhardt I, Holmes EA (2019). Sleep disturbance and intrusive memories after presenting to the emergency department following a traumatic motor vehicle accident: an exploratory analysis. Eur J Psychotraumatol.

[5] Kupfer DJ, Regier DA (2011). Neuroscience, clinical evidence, and the future of psychiatric classification in DSM-5. Am J Psychiatry.

[6] Creamer M, O’Donnell ML, Pattison P (2004). The relationship between acute stress disorder and posttraumatic stress disorder in severely injured trauma survivors. Behav Res Ther.

[7] Solberg Ø, Birkeland MS, Blix I, Hansen MB, Heir T (2016). Towards an exposure-dependent model of post-traumatic stress: longitudinal course of post-traumatic stress symptomatology and functional impairment after the 2011 Oslo bombing. Psychol Med.

[8] Ehlers A, Hackmann A, Michael T (2004). Intrusive re-experiencing in post-traumatic stress disorder: phenomenology, theory, and therapy. Memory..

[9] Bryant RA, Creamer M, O’Donnell M, Forbes D, McFarlane AC, Silove D (2017). Acute and chronic posttraumatic stress symptoms in the emergence of posttraumatic stress disorder: a network analysis. JAMA Psychiatry.

[10] Iyadurai L, Visser RM, Lau-Zhu A, Porcheret K, Horsch A, Holmes EA, James EL (2019). Intrusive memories of trauma: a target for research bridging cognitive science and its clinical application. Clin Psychol Rev.

[11] James EL, Bonsall MB, Hoppitt L, Tunbridge EM, Geddes JR, Milton AL, Holmes EA (2015). Computer game play reduces intrusive memories of experimental trauma via reconsolidation-update mechanisms. Psychol Sci.

[12] Holmes EA, James EL, Coode-Bate T, Deeprose C (2009). Can playing the computer game “Tetris” reduce the build-up of flashbacks for trauma? A proposal from cognitive science. PLoS One.

[13] Holmes EA, James EL, Kilford EJ, Deeprose C (2010). Key steps in developing a cognitive vaccine against traumatic flashbacks: visuospatial Tetris versus verbal Pub Quiz. PLoS One.

[14] Iyadurai L, Blackwell SE, Meiser-Stedman R, Watson PC, Bonsall MB, Geddes JR, Nobre AC, Holmes EA (2018). Preventing intrusive memories after trauma via a brief intervention involving Tetris computer game play in the emergency department: a proof-of-concept randomized controlled trial. Mol Psychiatry.

[15] Horsch A, Vial Y, Favrod C, Harari MM, Blackwell SE, Watson P, Iyadurai L, Bonsall MB, Holmes EA (2017). Reducing intrusive traumatic memories after emergency caesarean section: a proof-of-principle randomized controlled study. Behav Res Ther.

[16] Rothbaum BO, Kearns MC, Price M, Malcoun E, Davis M, Ressler KJ, Lang D, Houry D (2012). Early intervention may prevent the development of posttraumatic stress disorder: a randomized pilot civilian study with modified prolonged exposure. Biol Psychiatry.

[17] Gold SM, Enck P, Hasselmann H, Friede T, Hegerl U, Mohr DC, Otte C (2017). Control conditions for randomised trials of behavioural interventions in psychiatry: a decision framework. Lancet Psychiatry.

[18] Bird VJ, Le Boutillier C, Leamy M, Williams J, Bradstreet S, Slade M (2014). Evaluating the feasibility of complex interventions in mental health services: standardised measure and reporting guidelines. Br J Psychiatry.

[19] Eldridge SM, Lancaster GA, Campbell MJ, Thabane L, Hopewell S, Coleman CL (2016). Defining feasibility and pilot studies in preparation for randomised controlled trials: development of a conceptual framework. PLoS One.

[20] Electronic Arts Inc. Tetris [3.0.15.232402.1845.4685982517166080]. 2018

[21] Kanstrup M, Singh L, Göransson KE, Widoff J, Taylor RS, Gamble B, Iyadurai L, Moulds ML, Holmes EA (2021). Reducing intrusive memories after trauma via a brief cognitive task intervention in the hospital emergency department: an exploratory pilot randomised controlled trial. Transl Psychiatry.

[22] NVivo (2011). QSR International’s NVivo 11 qualitative data analysis Software.

[23] Smith J, Firth J (2011). Qualitative data analysis: the framework approach. Nurs Res.

[24] Gale NK, Heath G, Cameron E, Rashid S, Redwood S (2013). Using the framework method for the analysis of qualitative data in multi-disciplinary health research. BMC Med Res Methodol.

[25] Skene I, Pott J, McKeown E (2017). Patients’ experience of trauma care in the emergency department of a major trauma centre in the UK. Int Emerg Nurs.

[26] Berg LM, Florin J, Ehrenberg A, Östergren J, Djärv T, Göransson KE (2016). Reasons for interrupting colleagues during emergency department work – a qualitative study. Int Emerg Nurs.

[27] Berg LM, Källberg A-S, Ehrenberg A, Florin J, Östergren J, Djärv T, Brixey JJ, Göransson KE (2016). Factors influencing clinicians’ perceptions of interruptions as disturbing or non-disturbing: a qualitative study. Int Emerg Nurs.

[28] Källberg A-S, Ehrenberg A, Florin J, Östergren J, Göransson KE (2017). Physicians’ and nurses’ perceptions of patient safety risks in the emergency department. Int Emerg Nurs.

[29] Good AMT, Driscoll P (2002). Clinical research in emergency medicine: putting it together. Emerg Med J.

[30] Internetstiftelsen. Svenskarna och internet 2018 [Internet]. Svenskarna och internet. [cited 2021 Mar 22]. Available from: https://svenskarnaochinternet.se/rapporter/svenskarna-och-internet-2018/allmant-om-internetutvecklingen/smarta-mobiler-fortsatter-oka/

[31] Blackwell SE, Woud ML, MacLeod C (2017). A question of control? Examining the role of control conditions in experimental psychopathology using the example of cognitive bias modification research. Span J Psychol.

[32] Baddeley AD, Andrade J (2000). Working memory and the vividness of imagery. J Exp Psychol Gen.

[33] Singh L, Espinosa L, Ji JL, Moulds ML, Holmes EA (2020). Developing thinking around mental health science: the example of intrusive, emotional mental imagery after psychological trauma. Cognit Neuropsychiatry.

[34] Kanstrup M, Singh L, Göransson KE, Gamble B, Taylor RS, Iyadurai L, Moulds ML, Holmes EA (2021). A simple cognitive task intervention to prevent intrusive memories after trauma in patients in the emergency department: a randomized controlled trial terminated due to COVID-19. BMC Res Notes.

[35] Stein MB, Kerridge C, Dimsdale JE, Hoyt DB (2007). Pharmacotherapy to prevent PTSD: results from a randomized controlled proof-of-concept trial in physically injured patients. J Trauma Stress.

[36] Bragesjö M, Arnberg FK, Andersson E (2021). Prevention of post-traumatic stress disorder: lessons learned from a terminated RCT of prolonged exposure. PLoS One.

[37] Bragesjö M, Larsson K, Nordlund L, Anderbro T, Andersson E, Möller A (2020). Early psychological intervention after rape: a feasibility study. Front Psychol.

[38] Thabane L, Ma J, Chu R, Cheng J, Ismaila A, Rios LP, Robson R, Thabane M, Giangregorio L, Goldsmith CH (2010). A tutorial on pilot studies: the what, why and how. BMC Med Res Methodol.

[39] Levati S, Campbell P, Frost R, Dougall N, Wells M, Donaldson C, Hagen S (2016). Optimisation of complex health interventions prior to a randomised controlled trial: a scoping review of strategies used. Pilot Feasibility Stud.

