# Supplementary material for: Reaching people soon after a traumatic event: an exploratory observational feasibility study of recruitment in the emergency department to deliver a brief behavioral intervention via smartphone to prevent intrusive memories of trauma
Source: Pilot Feasibility Stud. 2021 Oct 7;7:184. doi: 10.1186/s40814-021-00916-x (PMC8499533; doi:10.1186/s40814-021-00916-x)
Supplement: Supplementary file 1 — Additional file 1. Procedure for collecting observational notes. Qualitative analyses - Framework method. [file 40814_2021_916_MOESM1_ESM.docx]

**Supplementary material**

**Procedure for collecting observational notes**

Observational notes were collected across the study period, and the form and content of notes were discussed several times among all members of the research team, to attune and ensure that the data collection was directed at the most relevant issues of uncertainty at the time. For instance, early on in the field work, observational notes were read within the team and discussed in order to cover key areas of uncertainty as these emerged from the observational data, e.g. when to best approach staff or a potential participant. Across time, the types of observational notes taken changed in accordance with the issues of priority at the time. The team developed a structured way of taking research notes so that they covered relevant information. Notes were taken by ER, LS (MSc students), MK (licensed clinical psychologist, PhD) and AR (registered nurse, PhD).

**Qualitative analyses - Framework method**

The framework method was used to conduct qualitative analyses in NVivo 11 Pro ([1](#_ENREF_1)). The framework method is an approach used within thematic analyses to manage and analyse qualitative data. It involves a sequence of interconnected steps to assist the researcher in structuring the data by moving back and forth across the data until a comprehensible interpretation develops ([2](#_ENREF_2)). The framework approach is often used in feasibility studies because it is appropriate when the purpose of the analysis is highly focused, and when researchers work with structured questions and aspire to undertake analysis systematically ([2](#_ENREF_2)). The method is executed in seven interrelated steps listed below ([2](#_ENREF_2), [3](#_ENREF_3)): In the first step, raw data is transcribed for analysis. In the second step, familiarization with the data is achieved by re-reading the transcript and documenting any reflections. In this study, researchers checked, anonymized and re-read the observational notes, adding their thoughts and impressions in the form of research memos. Particular attention was paid to hospital context and any deviant situations as we were keen to compare and contrast the reasons why members of the team had been especially successful or hindered in their attempts to recruit participants. The third step consists of coding of raw data (i.e. a process were data is given a descriptive label that is assigned to pieces of raw data). In this study, this ‘open coding’ process meant that everything that might be relevant for the study aim was coded line-by-line. Here the indexing and coding was guided by research questions 1 to 3: Recruitment, adaptation of procedures, and attitudes toward the study and research team. In the fourth step, an analytical framework is developed which involves grouping codes together into categories, thus creating a new structure for the data. In this study, researchers discussed which codes to include in the analysis and how to group them. Observational notes were initially analysed jointly (initial coding ER, LS, AR) and then final coding by AR to create a list of initial codes, which were then combined, refined and organized into a hierarchy of categories and sub-categories.

Diagrams were created based on the research questions in order to clearly define the relationship between different categories. Text relating to how recruitment procedures fitted around hospital routines was reviewed separately to form a base for descriptive summarizing results. In the fifth step, this analytical framework was applied to the rest of the data, i.e. the text was indexed and coded in accordance with the developed framework. We indexed the material using existing categories and codes from the diagrams. The use of the computer-assisted qualitative data analysis software NVivo ensured that this process was traceable and data easily retrievable at later stages. The sixth step consists of reworking, reducing and comparing the data with original observational text. In this study, the coded data was condensed and summarized based on the research questions. Because the study did not at this early stage have informed consent to publish data connected to specific individuals of the emergency staff we were not able to include references to illustrative quotations. The seventh and final step concerns data interpretation. In this study, the researchers discussed connections between each category to explore the overlap and relationships between codes and categories (AR, EAH, LS, ER, MB), and the final coding was reviewed within the full research team.

A combination of inductive and deductive coding was applied. In stage 3 an inductive approach was applied and observations were coded by so called “open coding” (i.e. coding the entire observation by tagging and naming selections of text). Here, frequent and significant codes and categories were identified. The inductive approach was applied to ensure that important aspects of the data were not missed. After stage 4 when the frameworks were developed, further coding was more deductive and guided by the specific research questions. This means that the sections of text within each data item that related to each research question was indexed, coded, analysed and described. The final codes and categories were closely related since for example staff attitudes to the study and study team were closely related.

The observational notes were transcribed to a total of seventeen documents, in which 127 observations were coded regarding identification of eligible patients and 87 observations were coded regarding staff attitudes. We also collected qualitative feedback from participants in the study via open ended questions asked verbally during their time in the ED, and from written responses to open-ended questions in the on-line follow-up assessments.

**References**

1. NVivo. QSR International's NVivo 11 qualitative data analysis Software.2011.

2. Smith J, Firth J. Qualitative data analysis: the framework approach. Nurse researcher. 2011;18(2):52-62.

3. Gale NK, Heath G, Cameron E, Rashid S, Redwood S. Using the framework method for the analysis of qualitative data in multi-disciplinary health research. BMC Med Res Methodol. 2013;13:117.
